# Maccabi proactive Telecare Center for chronic conditions – the care of frail elderly patients

**DOI:** 10.1186/s13584-017-0192-x

**Published:** 2017-12-11

**Authors:** Avi Porath, Angela Irony, Avital Segal Borobick, Shuruk Nasser, Ateret Malachi, Naama Fund, Galit Kaufman

**Affiliations:** 1 Maccabi Healthcare Services, 27 Hamered Street, 6812509 Tel Aviv, Israel; 2 Public Health Department, Faculty of health, Ben-Gurion University of the Negev, Beersheba, Israel

**Keywords:** Remote consultation, Health maintenance organizations, Frailty, Elderly patient

## Abstract

**Background:**

In 2012, Maccabi Healthcare Services founded Maccabi Telecare Center (MTC), a multi-disciplinary healthcare service providing telemedical care to complex chronic patients. The current paper describes the establishment and operation of the MTC center, from the identification of the need for the service, through the design of its solution elements, to outcomes in several areas of care.

We analyze the effects of the program on elderly frail patients, a growing population with complex and costly needs.

**Methods:**

Observational quasi-experimental analyses using propensity score matching was used to assess the effect of MTC’s operation on utilization outcomes including direct costs.

**Results:**

Results for frail elderly patients with complex chronic conditions show significant reductions in hospitalization days and hospitalization costs. MTC interventions also entailed lower overall average monthly costs in frail patients.

**Conclusion:**

We conclude that a proactive telehealth service for complex chronic patients using education, empowerment to self-management, and coordination of care is a cost-effective means of improving quality care and health outcomes in frail elderly patients.

**Electronic supplementary material:**

The online version of this article (10.1186/s13584-017-0192-x) contains supplementary material, which is available to authorized users.

## Background

The accelerated development of advanced technological solutions in the last decade holds promise for increased quality and access to health care [1]. Telecare, one such innovation, is currently extending its initially exclusive focus on acute conditions to chronic conditions [2]. Telecare and telehealth services allow personal, tailored care at up-to-date professional standards through coordination of care involving multiple providers and rapid response to changes in patients’ condition [3–5]. Elderly patients with advanced chronic conditions and a combination of physical and mental comorbidities and disability comprise a complex and challenging population for treatment: Their diverse clinical needs require substantial resources to prevent deterioration and prolonged hospitalization. Telecare is especially suitable for frail, home-bound patients, with complex medical conditions, and for patients in remote and rural areas where the knowledge and expertise that are associated with academic centers are less available [6, 7].

Frail, older patients pose such a system challenge as well as to the primary care physician who may often feel overwhelmed by their complex presentation and tenuous health status. Tools to identify frailty in the primary care setting are still in the preliminary stages of development [8]. Reports on the merits of telecare in frail elderly patients are limited. A systematic review of telecare in elderly frail patients found only few studies that were not limited to specific condition and that only 1% of them met the inclusion criteria of randomized studies or observational studies with more than 80 patients [9]. The authors concluded that proactive support from practitioners by telephone can improve clinical outcomes and that the cost-effectiveness of these interventions was less certain.

In this observational study we use quasi-experimental methods to compare the effects of the MTC program on frail elderly patients recruited in 2015 to similar patients in MHS registry.

## Maccabi Telecare service

Maccabi Healthcare Services (MHS) is the second largest and fastest growing HMO in Israel (controlling 25% of the Israeli HMO market). It is a non-profit mutual health fund that provides healthcare to more than 2 million members. MHS’s population has aged rapidly in the past two decades and the subpopulation of 65+ is expected to account for 14% of all MHS members by 2025.

MTC is a multi-disciplinary healthcare center established by MHS in July 2012 to provide telemedical care to complex chronic patients. It is staffed by a multi-disciplinary team of health practitioners including nurses, consulting physicians, clinical pharmacists, social workers and nutritionists. MTC currently serves approximately 6000 patients, and has rendered services to over 22,000 patients since the inception of the service. This paper focuses on the 389 frail elderly patients that were recruited to the MTC service in 2015.

The basic aims of the MTC center are to provide high-quality proactive home-based telecare for complex chronic patients through education, empowerment to self-care, and coordination between various care providers, leading to increased adherence to treatment and an eventual decline in the use of emergency services and hospitalization rates.

The MTC Center responds to members’ inquiries 24/7, independent of patients’ geographic location. Each patient is assigned a personal nurse who proactively conducts medical monitoring of the patient’s condition to prevent complications through early detection of changes and real-time interventions. The team operates in full collaboration with primary care physicians and other healthcare practitioners across a variety of treatment facilities.

A key component of the solution developed by MHS is the use of a remote telecare system who’s organizing principles and work procedures are described in the Additional file 1: Appendix. A short description of the roles and responsibilities, use of online protocols, work procedures and information and communication technologies is given in this section.

### Roles and responsibilities

#### MTC personal nurse (MPN)

The MPN is the patient’s care integrator. The MPN proactively contacts each assigned patient, gathers information and develops a personalized care plan including clinical targets, which is approved by the primary physician.

#### Primary care physician (PCP)

The community-based family physician is the patient’s case manager. The PCP approves the patient’s referral to MTC, approves the MTC intervention plan, provides and revises prescriptions, writes referrals, and is informed by the MPN of developments related to the patient.

#### The MTC *team*

Includes several types of physician consultants (cardiologists, pulmonary specialists, endocrinologists, and psycho-geriatricians), clinical pharmacists, social workers, nutritionists and an administrative team.

### Guidelines and protocols

Protocols and guidelines are valuable tools for promoting evidence-based medicine, treatment safety, reduce risks, and increase standardization of service. The digital protocols (for example, protocols on changing drug therapy) were integrally incorporated into Electronic Medical Record (EMR) and are visible to all caregivers. Deviations and adaptations of protocols are documented for discussions on further service modifications and improvements and periodic evaluations.

### Work procedures

#### Enrollment

Potential patients in each treatment field are identified by a computerized system algorithm (CSA) that runs continually on the MSH database and IT system. The patient’s PCP is alerted through the EMR of the patient’s eligibility for MTC and is asked to approve the patient’s enrollment in the service. Approved patients are invited by telephone to join MTC, and formal consent is solicited at that time.

#### Routine follow-up and monitoring

Using online protocols the MPN continuously and proactively monitors the patient’s condition, provides guidance and empowerment, supports the patient and his/her care giver, and assists the patient’s PCP.

#### Coordination/ cooperation with PCP

The PCP receives quarterly reports of the patient’s condition and is informed promptly of any change in the patient’s condition.

### Information and communication technologies

This integrative service combines and coordinates MHS’ EMR and CRM systems with online clinical protocols to assure online team collaboration with caregivers and efficiency of scheduling calls and follow-up. Information derived from telecare devices, such as tablets and transmitting glucometers are also integrated into the operating system. Finally, an analytical system generates managerial reports used to optimize operations and analyzes patient outcomes.

## Frail patients in MHS

Inclusion in the MTC intervention program for frail elderly patients required that patients met two or more of the following criteria: adults with two or more ER visits or hospitalizations per year; three or more active chronic diseases; polypharmacy (purchasing six or more medications); and albumin serum level < 3.3 g/dl, difficult ambulation as determined by community nurses, otherwise independent. Exclusion criteria included oncological patients, major psychiatric illnesses and difficulties in communication.

Eligible patients identified by a computerized system algorithm were entered into the MHS Frail Elderly Patient Registry and an alert popped-up in their EMR notifying their PCP of their eligibility for the MTC service. After approving a patient for referral to MTC, the PCP obtained patients’ written informed consent (giving the MTC team permission to contact the patient according to protocol). Preferred modes of communication between MTC, PCPs, and patients were established. All unenrolled patients continued to receive standard care in the community.

## Data

Information on all MHS members is stored in a large central computerized database. The database includes information regarding comorbidities, hospitalizations, emergency department visits, physician visits, outpatient specialist visits, and purchase of medications, laboratory test results. Patient data can be retrieved from this database.

## Methods

We assessed the outcomes of MTC comparing the following one-year outcomes to the corresponding pre-recruitment period for each MTC patient: hospitalization days, service utilization, and average monthly costs.

Descriptive statistics of patients were calculated and expressed as means and standard deviations (SD) for continuous variables and as numbers and percentages for dichotomous variables.

As the MTC service was offered nationwide but the decision to refer patients to the service was left to the discretion of primary care physicians, we could not assume that MTC patients and unenrolled MTC candidates (control patients) were comparable. Therefore, we used the propensity score matching (PSM) method to evaluate the differences between MTC and control patients. To match control patients to MTC enrollees, a logistic regression model was applied to calculate the probability of MTC enrollment based on gender, age, number of comorbidities, socio-economic status and costs in the 12 months prior to the recruitment. Outcomes of propensity-matched control patients were retrieved from the relevant disease registries and organizational costs databases.

Average monthly costs during the distant 6 to 12 months prior to recruitment (not the 6 months immediately preceding recruitment) were also used in the PMS for the economic analysis. The matching of costs with the distant 6 months was done to minimize the potential effect of escalating cost in the months immediately prior to recruitment to MTC. After calculation of their propensity scores, the effect of telecare on medical expenditures of the two groups was estimated using the difference in difference method [10]. We compared the direct medical annual costs of patients in both groups during the year following the recruitment to MTC with the preceding year.

The propensity score matching procedure for SPSS (version 3.0.2, programmed by Felix Thoemmes) [11] using the caliper method was used to match patients based on their probability to enroll in the MTC. A chi-square test for categorical variables and two-sample t-test for continuous variables were performed to determine significant differences in baseline characteristics and changes between groups. Multivariate regression models were applied to test the statistical significance of the difference in hospitalization days and costs between intervention and control groups, adjusted for potential confounders. Statistical significance was defined as *p* < 0.05. All analyses were conducted using standard statistical software (SPSS version 22, Inc., Chicago, IL).

## Results

The intervention group comprised 389 frail elderly patients who enrolled in MTC in 2015. The remaining 6068 unenrolled MTC candidates comprised the potential pool from which the control group was extracted based on a propensity score algorithm. The analysis thus included 388 matched pairs of frail patients. Table 1 presents profiles of the study sample and their matched controls.

**Table 1:** Profile of frail elderly patients, 2015

Variable	Registry Pool	MTC	Matched Controls	*p*-value*
Patients	6068	388	388	
% male	46.4	44.8	43.0	N.S.
Age mean (s.d.)	81.6 (6.5)	79.6 (7.4)	79.6 (7.2)	N.S.
Range	60.1–103.8	60.3–98.0	60.1–101.1	
Comorbidities mean (s.d.)	3.8 (1.3)	4.1 (1.3)	4.0 (1.4)	N.S.
Charlson’s score mean (s.d.)	3.7 (3.0)	4.0 (2.8)	3.7 (2.8)	N.S.
% Diabetes	58.3	61.3	62.6	N.S.
% Hypertension	94.0	94.6	93.8	N.S.
% IHD	43.4	52.3	47.9	N.S.
% CVA/TIA	25.6	27.1	28.9	N.S.
% Heart failure	6.2	11.6	8.2	N.S.
% COPD	9.5	12.6	9.5	N.S.
% Dementia	22.2	19.3	22.2	N.S.
Socioeconomic status (% low)	19.3	20.4	22.2	<0.01

*Comparing MTC and matched control patients

The study sample was predominantly female, average age 80, with four comorbid physical conditions on average. The prevalence of individual chronic conditions as well as the Charlson’s comorbidity score [12] reflect these patients advanced age. One fifth of the patients were classified as “low” socioeconomic status (score 1–4 out of 10) based on neighborhood residence documented in Maccabi’s database and the census data conducted by the Central Bureau of Statistics in 2008.

### Utilization of services

Table 2 presents average monthly utilization data and costs, comparing the intervention and the matched control group in the 12 months preceding and following the intervention. Compared to the matched controls, frail elderly MTC patients had a higher pre-intervention rate of hospital days, ER visits, and costs. In the intervention, monthly rates of utilization and costs of MTC patients declined while hospital days and costs increased in the control group. Pre- and post-intervention differences between groups in hospital days, ER visits and costs were statistically significant.

**Table 2:** Utilization data

Variable	MTC	Control	*p*-value*
Patients	388	388	
Hospital days, mean (s.d.)			
pre-intervention	0.48 (0.76)	0.32 (0.93)	< 0.05
post-intervention	0.37 (0.97)	0.43 (1.15)	
ER visits, mean (s.d.)			
pre-intervention	0.07 (0.09)	0.04 (0.08)	< 0.05
post-intervention	0.05 (0.09)	0.03 (0.06)	
PCP visits, mean (s.d.)			
pre-intervention	1.84 (1.1)	1.55 (1.0)	N.S.
post-intervention	1.80 (1.2)	1.44 (1.0)	

*p values compare the differences between groups for the pre-post changes

Multivariate analysis controlling for age, gender, prior year hospitalization days, number of comorbid conditions, and socioeconomic status assessed the impact of MTC on days of hospitalization. The strongest predictor of hospital days in the intervention year was previous year’s hospital days (*β* = 0.112, *p* < 0.01). MTC was associated with a reduction in hospital days (*β* = −0.04, *p*-value <0.05).

### PCP visits

**Fig. 1:**
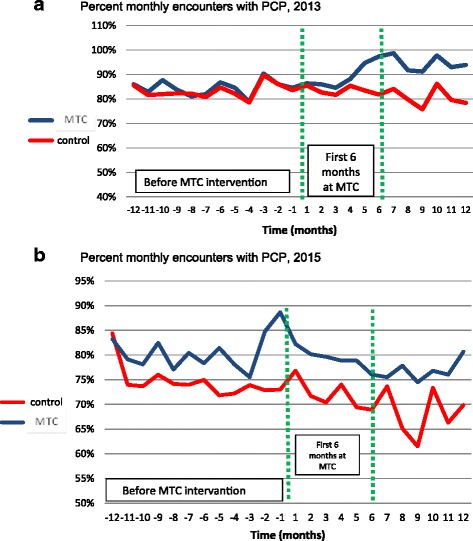
**a**: Percent monthly encounters with PCP, 2013. **b**: Percent monthly encounters with PCP, 2015

One of the problems of frail elderly patients is a gradual decline in the rate of encounters with a PCP. Figure 1 shows the trend over time of the proportion of patients who visited their PCP at least once a month in 2013 (1a) and in 2015 (1b).

In the 2013 cohort, MTC patients increased their rate of contact with PCP while the control patients did not. This phenomenon is less prominent in the 2015 cohort, and the change in average number of monthly visits to PCP between groups (excluding the tele-encounters with clinicians at the MTC) was not statistically significant (see Table 2).

### Mortality

Twelve of the MTC frail patients (3.1%) and 19 of the matched controls (4.9%) died during the first 12 follow-up months. This difference did not reach statistical significance.

### Monthly cost

**Fig. 2:**
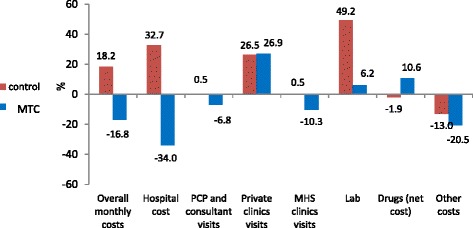
Percent change in average monthly cost, by costs components

Figure 2 shows the change in average monthly cost of MTC and control patients in the first 12 months of the intervention period compared to the preceding 12-month period. The cost data include the cost of the MTC intervention. Findings show a 17% reduction in overall monthly costs in MTC patients in contrast to an 18% increase in control patients. Hospitalization cost was the main contributor to the change in overall costs. Medication costs increased in MTC patients, mainly due to increased adherence.

Using multivariate analysis on costs controlling for age, gender, prior year monthly average costs, number of comorbid conditions and socioeconomic status the strongest predictor of monthly costs in the intervention period was previous period costs (*p* < 0.001); MTC was associated with a reduction in overall monthly costs (*p* < 0.001). A difference in difference analysis of annual costs prior to enrollment and one year post enrollment of the propensity matched groups showes a 15% reduction in the cost of frail MTC patients compared to an increase of 13% in the matched grouo (*p* < 0.05).

## Discussion

Comparing the MTC to other large scale telecare programs is challenging. These programs differ in the choice of target populations, the level of comprehensiveness of the interventions, mode of communication with patients and among caregivers and in the level of integration with the organizations’ IT systems. Here are some examples. The large British Whole System Demonstrator (WSD) program used cluster randomization and required participant PCP to operate two systems of reporting. Economic analysis used administrative data. One study concluded that telehealth did not seem to be a cost effective addition to standard support and treatment [13] while others reported lower mortality and ED admissions in the telecare group [14].

Research published by York University in 2009 evaluating the impact of the Scottish Joint Telecare Project identified indicative cost savings as a result of implementing a coherent telecare program [15].

A review of the Veterans Administration funded telecare studies that used different technologies of communications with patients concluded that the sustainability of telemedicine interventions for the broad spectrum of veteran patient issues and the ongoing technology training of patients and providers are challenges to telemedicine-delivered care [16]. Meta analytical studies on cost effectiveness of telecare programs are inconclusive [17, 18].

The current paper introduces the rationale, operations and implementation of MTC, a tele-based chronic care service established by MHS. As this service is offered to a large number of patients, its evaluation uses quasi-experimental methods. As an illustration of its outcomes, we examined the effect of MTC on frail elderly patients with complex chronic conditions. A significant benefit was achieved in these older-old patients, specifically a reduction in hospitalizations and overall costs.

The telecare service was desined to serve a large number of complex patients to supplement concurrent community services. We offer the service to consenting patients for approximately 6 to 12 months in order to assure accessibility to new patients. Since the service is offered free of charge to patients, the financial aspects of its operation are extremely important to its sustainability, and reduced hospital costs during the interrvention is a major determinant of the positive ROI for some patients groups and for the overall program affordability. As MHS is a non-profit organizations savings in management of one group of patients serves to offer telecare services to other groups of patients who benefit clinically at additional costs to MHS such as cancer patients during their active treatment period and stoma patients.

The architecture of the program ensures that PCPs are informed of all decisions made by MTC, and remain in charge of prescribing medication: Therefore PCPs are under no threat of loss of patients to an external program (with corresponding loss of income). Consultant physicians that were part of the team consulted the PCPs assuring the valuable continuity of care. These features of the program contributed to its successful uptake and to both patients’ and PCPs’ high satisfaction with the program. The large geographical distribution of patients was another consideration in favor of keeping the PCPs as part of the team. Yet the challenge of communicating with hundreds of PCPs may be great for the coordinating staff as compared with a few full-time program consultant doctors with whom they are in constant communication. Despite the key importance of this distinction (keep the PCP/replace the PCP), we could not find any study that compared the two approaches, either using empirical data or simply as a thought exercise. We believe that this is an important distinction that deserves further study.

Another unique feature of the program is its data-driven algorithms that identifis qualifying candidates patients and alerts their PCPs using the EMR system. This “proactive” method of recruitment contributed to its egalitarean and comprehensive spread that was limited only by the system capacity.

As all activities involving MTC patients are shared through the patients’ integrated EMRs, there was no loss of data nor the inconvinience for the clinicians to leave their familiar EMR and use another operating system.

The integration of clinical data with operating data and costs enable periodic evaluation of various aspects of the service including its cost-effectiveness. Three consequent annual rounds of assessment have confirmed the value of the telecare services for several groups of patients. The assessment procedure ensures that gradual modifications are continually introduced to maximize cost-effectiveness.

One limitation of this intervention program is its capacity, which is currently limited to several thousand patients per year, due to the need to train and recruit personnel and deploy the service to a large number of PCPs. A second limitation of the program is the population-based and observational mode of evaluation, which uses a quasi-experimental design of data analysis. Propensity score analysis on enrollment probability was used to select controls from the potential pool of registry patients. Nevertheless, residual confounding may be significant and weaken the strength of our conclusions.

## Conclusions

In conclusion, large-scale, proactive, coordinated, home-based telecare services for elderly frail patients using personalized clinical protocols together with patients’ training and empowerment to self-management is cost-effective and can improve outcomes.

## Additional file

Additional file 1: Appendix. Maccabi Telecare Service. (DOCX 24 kb)

## Abbreviations

CHF: Congestive Heart Failure
COPD: Chronic Obstructive Pulmonary Disease
CRM: Customer relationship management
CSA: Computerized System Algorithm
CVA/TIA: Cerebrovascular accident / transient ischemic attack
DSS: Decision Support System
EMR: Electronic Medical Record
ER: Emergency Room
ESC: European Society of Cardiology
GOLD: Global Initiative for Chronic Obstructive Lung Disease
HMO: Health Maintenance Organization
IT: Information Technology
IVR: Interactive Voice Response
MHS: Maccabi Healthcare Services
MPN: MTC Personal Nurse
MTC: Maccabi Telecare Center
NYHA: New York Heart Association
OTC: Over the Counter
PCP: Primary Care Physician
PSM: Propensity Score Matching
ROI: Return on Investment
SD: Standard Deviation
WSD: Whole System Demonstrator
