# Supplementary material for: Maccabi proactive Telecare Center for chronic conditions – the care of frail elderly patients
Source: Isr J Health Policy Res. 2017 Dec 11;6:68. doi: 10.1186/s13584-017-0192-x (PMC5724333; doi:10.1186/s13584-017-0192-x)
Supplement: Additional file 1: — Appendix. Maccabi Telecare Service. (DOCX 24 kb) [file 13584_2017_192_MOESM1_ESM.docx]

**Appendix**: **Maccabi Telecare Service**

MTC is a multi-disciplinary healthcare center established by Maccabi Healthcare Services in July 2012 to provide telemedical care to complex chronic patients. It is staffed by a multi-disciplinary team of health practitioners including nurses, consulting physicians, clinical pharmacists, social workers and nutritionists. MTC currently serves approximately 6,000 patients, and has rendered services to over 22,000 patients since the inception of the service. It operates in full collaboration with primary care physicians and other healthcare practitioners across a variety of treatment facilities. MTC’s unique service is based on five principles: a proactive care approach, availability and accessibility; advanced technologies; integrated and coordinated care; and multi-disciplinary teamwork.

MTC provides care according to the chronic care model via telemedicine. Each patient is assigned a personal nurse who proactively conducts medical monitoring of the patient’s condition to prevent complications through early detection of changes and real-time interventions. Care is delivered by a multi-disciplinary team that provides maximal accessibility and availability: The MTC Center responds to members’ inquiries 24/7, independent of patients’ geographic location.

MTC was established to address two issues: (a) to answer the discrepancy between the needs of complex chronic, multi-morbid, disabled patients and the services provided by community clinics or hospitals , which leads to sub-optimal clinical outcomes, low patient satisfaction, and inefficient resource use; [and (b)](http://search.proquest.com/health/indexinglinkhandler/sng/au/Lee,+Thomas+H/$N?accountid=33654)  limited availability of financial and human resources along with the prospect of an aging membership of MHS, including a growing subpopulation with complex needs and high costs.. The basic aims of the MTC center are to provide high-quality proactive home-based telecare for complex chronic patients through education, empowerment to self-care, and coordination between various care providers, leading to increased adherence to treatment and an eventual decline in the use of emergency services and hospitalization rates. A key component of the solution developed by MHS is the use of a remote telecare system who's organizing principles and components are presented in the next section.

**Solution Elements**

Population

In targeting populations for intervention, priority was given to patient populations that show increased use of medical services over time. Treatment fields were also selected where potential for cost savings was identified. Inclusion and exclusion criteria were defined, and a computerized system was developed on the basis of an algorithm that scans organizational central databases and identifies information concerning these potential populations, including population size and total cost of care. Clinical conditions were distinguished by their need for short-term or long-term treatment interventions. Inclusion of new fields of care is considered periodically on the basis of the scientific literature, global expertise, the detection of geographical gaps in service, and the availability of support systems. Table 1 presents the 2015 criteria and size of MTC treatment groups by treatment field and intervention type.

Table 1: *MTC Intervention Inclusion Criteria and Population Size, 2015*

| Intervention duration | Clinical conditions | Inclusion criteria | N |
| --- | --- | --- | --- |
| Long-Term  Interventions | CHF | NYHA Class II, III | 1,131 |
|  | COPD | GOLD 2, 3 | 994 |
|  | Homecare Unit | Patients in a stable condition who are under the care of the Homecare unit due to a chronic disease | 715 |
|  | Frail elderly patients | Patients with comorbidities and worsening of their chronic disease and function | 1,563 |
| Short-Term  Interventions | Diabetes | Type 2 patients who recently began treatment with insulin and/or diabetes is uncontrolled | 721 |
|  | Oncology | Patients receiving active treatment (chemotherapy/radiation therapy/biological therapy) | 745 |
|  | Stoma | Patients after recent colostomy | 102 |
|  | Hard-to-heal wounds | Patients with hard-to-heal wounds | 31 |
| Total |  |  | 6,002 |

Guidelines and protocols

Protocols and guidelines are valuable tools for promoting evidence-based medicine, treatment safety, reduce risks, and increase standardization of service. At telemedicine centers, the use of online protocols is crucial due to the limitations of the medium (the inability to use sight or touch). In order to define a high standard of care, a multi-disciplinary work group developed clinical protocols for routine and emergency situations in each treatment field on the basis of international clinical guidelines specific to each field (i.e., the GOLD standard for COPD, or ESC for CHF). These protocols were subsequently validated by specialists and by the MSH risk management department. The digital protocols (for example, protocols on changing drug therapy) were integrally incorporated into Electronic Medical Record (EMR) and are visible to all caregivers. Deviations and adaptations of protocols are documented for discussions on further service modifications and improvements and periodic evaluations. Once a year, a steering committee reviews the needs in each field of care considering recent developments, and considers modifications to the services.

Roles and responsibilities

*MTC Personal Nurse (MPN).* The MTC personal nurse (MPN) is the patient's care integrator. The MPN proactively contacts each assigned patient, gathers information and develops a personalized care plan including clinical targets, which is approved by the primary physician. According to each customized plan, the MPN instructs the patient about his or her condition/s, potential complications, and an appropriate diet; directs the patient to perform daily activities; empowers him/her to self-management and prevention; monitors the patient's indices to detect potential deterioration; and initiates immediate interventions in response to changes in the patient’s condition.

*Staff manager / team lead*. The team leaders are nurses who have managerial authority. The duties of this role include team management, quality care, control of operational measures, and services.

*Primary Care Physician (PCP):* The community-based family physician is the patient's case manager. The PCP approves the patient's referral to MTC, approves the MTC intervention plan, provides and revises prescriptions, writes referrals, and is informed by the MPN of developments related to the patient.

*Physician consultant*: Several types of specialists (primarily cardiologists, pulmonary specialists, endocrinologists, and psycho-geriatricians) support the routine work of the multi-disciplinary team through medical consultations, especially in urgent and complex events, and also provide telephone consultations to the PCP and the MTC staff.

*Clinical pharmacist:* The clinical pharmacist provides pharmacological consultations to the MPN and the PCP, adjusts drug therapy for complex chronic patients (to reduce side effects and prevent drug interactions), and works to reduce inappropriate polypharmacy.

*Social worker*: The social worker provides consultations to support the staff on complex cases; provides a response to patients in distress or at risk, and helps patients exercise their rights.

*Nutritionist*: The nutritionist provides nutritional consultations to the staff on complex cases; tailors a personal nutritional program to each patient; and empowers patients by providing tools for nutritional self-management.

*Administrative team*: Specially-trained secretaries provide administrative assistance to patients, schedule clinic appointments and checkups.

*MTC manager*: The MTC manager supervises organizational processes through operative measures, quality care, and service delivery. The manager also performs periodic assessments of target attainment and economic evaluations of new services, and continually seeks to identify opportunities for service improvements.

*Patients*: Patients are encouraged to participate actively in the treatment process, comply with MPN guidance, and adhere to the medical regimen and self-monitoring.

Work procedures

The following work procedures were developed to ensure efficient operations of the MTC center:

*Enrollment.* This is the process through which patients from the community enter the service. First, potential patients in each treatment field are identified by a computerized system algorithm (CSA) that runs continually on the MSH database and IT system. Second, potential patients’ suitability for the service is assessed by the team lead in coordination with the patient’s PCP, after the PCP is alerted through the EMR of the patient's eligibility for MTC and is asked to approve the patient’s enrollment in the service. In addition, family physicians are free to recommend including patients that were not allocated by the algorithm. Third, the patient is invited by telephone to join MTC, and formal consent is solicited at that time. Finally, the MPN contacts the patient, introduces the service and its operations, documents the patient’s contact information, and confirms the patient’s consent to the service terms.

*Routine follow-up and monitoring*. This is the process in which proactive telephone calls are made to the patients, according to MTC protocols. Through these communications, the MPN and the patient establish a patient-caregiver relationship. The MPN continuously and proactively monitors the patient’s condition, provides guidance and empowerment, supports the patient and his/her care giver, and assists the patient's PCP.

The nurse-patient ratio at MTC is 1:250 (full-time nurses), although ratios vary by level of nursing experience and patient characteristics. Approximately 85% of the total incoming and outgoing calls at MTC are proactive outgoing calls, while 15% are incoming calls from patients.

*Coordination/ Cooperation with PCP*. The PCP is the patient’s physician of record or primary physician, who provides and revises prescriptions. Therefore, before a patient enters the MTC service, the patient’s PCP confirms the patient’s suitability for the service. The PCP receives quarterly reports of the patient’s condition and is informed promptly of any change in the patient's condition.

*Working in multi-disciplinary team*. The MTC team includes nurses, physician consultants, clinical pharmacists, social workers, and nutritionists. The MPN involves the team members as necessary.

*End of Service*. In treatment fields designated for short-term interventions, the period of service is between 6 and 9 months. Patients in the long-term intervention fields who reach their treatment targets before the end of this period (i.e., diabetes is controlled) may also be released from the program. Other causes of exit from the program include non-compliance, the occurrence of another severe disease, or prolonged hospitalizations. The patient's PCP is involved in the service termination decision, and such decisions are communicated to the patient by the MPN.

*Non-clinical triage*. Specially-trained secretaries receive incoming calls and perform initial triage by distinguishing between administrative and clinical calls. Administrative calls are handled by the secretaries, who assess them for their urgency and refer them to the MPN, as needed.

*Supervision*. Supervision is performed in two levels: real-time shift monitoring and retrospective through weekly reports. Performance of all MTC nurses is measured on several elements: (a) Productivity - Nurses are required to maintain a certain level of productivity, measured as the number of outgoing calls per hour/per shift; (b) Quality of care - Nurses are required to meet a high standard of treatment. Team leads engage in proactive quality control by listening to calls and reviewing EMRs. After each quality control action, the nurse receives feedback from the team lead.

Information and communication technologies

MTC patient care and follow-up is feasible due to several systems supported by information and communication technologies.

*Operating System*

To identify potential patients, the MTC Nurse system performs a data run every night involving data retrieval from several MHS data systems, including those covering hospitalizations, medications, and indicators of chronic disease. An advanced screening procedure assigns patients to specific nurses according to treatment field.

The system is completely interoperable with the EMR (see below), the chronic disease registries, and other systems, and enables the staff to maintain contact with approximately 1,000 PCPs. The system contains three modules:

- Population view (patients, hospitalized patients, prioritized tasks, etc.);
- Patient view (patient details); and
- Task view (task name, priority, target date, patient, etc.).

*Electronic Medical Record (EMR)*

This system is used to record and display medical information, and provides views of daily tasks and links to protocols and tasks. The MTC Nurse system is integrated into MHS's centralized EMR, which means that it is accessible by all care providers at MHS, who may either enter information or view updates regarding a patient's condition, thus ensuring information continuity.

An evidence-based Decision Support System (DSS) integrated within the EMR displays tailored guidelines on responding to medical events, interview scripts based on medical logic (used by MPNs when communicating with patients) and a rapid OTC dosage calculation engine.

*Unified Communication System*

This system allows MTC providers to conduct audio and video calls with patients. It is fully integrated with IVR (Interactive Voice Response) and the organization’s information systems. All calls are recorded for quality control and training purposes. Incoming calls are automatically routed according to the patient’s assigned MPN.

*360^0^ Management Tools*

The management team uses an analytical system and an optimization system to analyze, manage, and evaluate populations and operations. The analytical system combines information from a large number of systems and performs a 360^0^ analysis. The system generates managerial reports used to manage operations and analyzes patient populations. The reports provide information on various aspects of patient care and MPNs' performance, related tasks, clinical information, information on resources invested per patient, statistics of hospitalizations, service consumption, etc.

The optimization system is based on a methodology that performs a professional, in-depth analysis of operations. Results are displayed on an easy-to-use dashboard that enables users to accurately identify areas of concern in real time, such as poor performance, and make decisions quickly to take focused action to improve efficiency. A drill-down inquiry on a range of parameters is feasible, allowing views by call, hour, or nurse.

Medical devices

Electronic devices play an integral role in MTC's follow-up procedures. The following three tele-medical sensors are currently given to patients for use at home, and deliver objective data on patients' medical indicators and behaviors, allowing MTC practitioners to monitor and assist in improving adherence to treatment in real time:

- *Tablets*, which are used by patients to enable face-to-face communication and facilitate evaluation of clinical status (such as wounds).
- *Transmitting glucometers* – an innovative app transforms the patient's mobile phone into a blood-sugar level analysis unit. Results are transmitted directly from the patients' phones to their EMR.
- *Electronic pill organizers* that transmit information on medicine use, time of consumption, and errors in consumption.
